# 

**Published:** 1843-01-21

**Authors:** 


### THE MEDICAL EXAMINER,

### And Retrospect of the Medical Sciences.

EDITED BY

MEREDITH CLYMER, M.D.

VOL. VI.] PHILADELPHIA, JANUARY 21, 1843. [No. 1.

